# Conditions for parents' participation in the care of their child in neonatal intensive care – a field study

**DOI:** 10.1186/1471-2431-8-3

**Published:** 2008-01-23

**Authors:** Helena Wigert, Anna-Lena Hellström, Marie Berg

**Affiliations:** 1 Institute of Health and Care Sciences, The Sahlgrenska Academy at University of Gothenburg, Göteborg, Sweden

## Abstract

**Background:**

To promote participation by parents in the care of their child in neonatal intensive care units (NICU), health professionals need better understanding of what facilitates and what obstructs participation. The aim was to elucidate conditions for parents' participation in the care of their child in NICUs.

**Methods:**

A field study with a hermeneutic lifeworld approach was used and data were collected at two NICUs through participative observations and interviews with representatives of management, staff and parents.

**Results:**

The results point to a number of contradictions in the way parents were offered the opportunity to participate in neonatal intensive care. Management and staff both had good ambitions to develop ideal care that promoted parent participation. However, the care including the conditions for parental participation was driven by the terms of the staff, routines focusing on the medical-technical care and environment, and budgetary constraints.

**Conclusion:**

The result shows that tangible strategies need to be developed in NICUs aimed at optimising conditions for parents to be present and involved in the care of their child.

## Background

It is a major challenge for health care professionals, such as nurses and physicians, to support participation by parents in the care of their child in neonatal intensive care units (NICU), and it raises the central question of how parents are invited to participate in this environment.

Parents who have a child in a NICU are vulnerable, they have not yet established a relationship with their child, and their treatment by health care professionals is significant [1]. The mother-child relationship is central to the development of the child [2-6], but mothers of children treated in a NICU have been found to feel left out, neither belonging to the maternity ward nor the neonatal unit, a feeling that is still present years afterwards [7]. Research shows that parents who are kept informed and are supported to take an active part in the care of their child in a NICU may gain a feeling of control of the situation, strengthening their parental identity [1,8,9]. The parents appreciate and trust medical competence and have a need to communicate with the staff concerning the care of their child [10].

Modern care in NICUs is based on parental care of the child when the child is an inpatient. To support this, Swedish mothers and fathers can receive economic compensation for loss of earnings, allowing them to stay in the hospital with their child [11]. A previous study, however, has shown that they have not been offered the right conditions to participate in the care of their child at the NICU. The staff was ambivalent, set limits and dictated conditions for parental participation [12]. The conditions have thus not been in place to meet the standards of the United Nations Children's Convention [13] and the Nordic Association for Sick Children in Hospital [14], which stress that staff should encourage presence and active participation by parents in the care of their child both day and night. In family-centred care the focus is on parental participation, which means that the parents should follow their child's care and that there should be a partnership between staff and parents [15-17]. Although family-centred and integrated care of the mother and child is more frequent today; it is not common [18].

In order to promote participation by parents in their child's care, it is therefore necessary to improve understanding of what facilitates and what obstructs this participation. The aim of this study was to elucidate conditions for parents' participation in the care of their child in a NICU. Here, participation includes physical presence as well as active partaking in the child's care.

## Methods

This was a participative, observational study that included interviews with staff and parents. It adopted a hermeneutic lifeworld approach, which offered the researcher a basis from which to analyse the world as experienced and communicated by people. The lifeworld is the everyday world in which we live our lives and take all our activities for granted. The research begins with tangible descriptions of lived everyday life experiences [19]: in this study the conditions for parents' participation in the care of their child in neonatal intensive care. The hermeneutic philosophy highlights that being in the world, and its interpretation is the basis of understanding, and language is an essential tool as it gives us access to other people's experiences [20]. Hermeneutic lifeworld research requires the researcher to have an open and sensitive attitude to the phenomenon being focused on, and it bridles pre-understanding through a distancing and reflective attitude to new experiences [19].

### The context

The study was conducted at two specially selected NICUs: one at a university hospital and one at a regional hospital in a smaller city. Both hospitals were located in the same Swedish region, implying similar political and financial management systems. The NICU at the university hospital admitted seriously ill children from other regional hospitals and had a high throughput of patients, often leading to a high workload. Once a child was in a more stable medical condition, he/she was transferred to another unit. The staff consisted of paediatric nurse assistants, nurses, physicians and administrators. The NICU at the university hospital had 22 beds and a staff of 120, and the local NICU had 15 beds and 60 staff. Common reasons for treating children were prematurity, breath dysfunction and infection. The durations of hospital stays at these two units varied from a few hours to several months, with a mean period of 13 and 8.2 days respectively.

### Ethics

Permission to perform the study was requested from the heads of the ward, and ethical approval and permission to undertake the study was requested from the Research Ethics Committee. The staff at the two selected NICUs was given verbal and written information about the study, and the interviewed staff and parents were personally informed. All interviewees were assured that participation was voluntary, that all information would be treated confidentially and that the tape-recorded and transcribed interviews would be locked securely in a fireproof place.

### Data collection

Data were collected over eight months in 2006 through participative observations (O) and interviews with staff and parents. The observations were directed at the phenomenon, i.e., conditions for parents' participation in the care of their child in NICUs, and were included to identify both facilitating and obstructing factors. The observations gave access to interpersonal interactions, and the combination of observations and interviews provided an insider's perspective on the phenomenon in its natural setting [19]. The data collector (HW) was a paediatric nurse with work experience from NICUs and, together with the results of previous studies [7,12], this influenced her pre-understanding. The intention, however, was to keep an open mind and to be ready to see, interpret and understand something new in a new way [20], and to be aware of the self in relation to the phenomenon being studied [19] through reflection on personal pre-understanding.

The fieldwork was carried out over 64 hours during 22 different working shifts. During the observations, the focus was on the staff's invitation or lack thereof to parents to participate in their child's care. The data collector's role was to become a member of the ward's working team, while at the same time allowing for reduced participation in activities when observations were being made. The data collector did not participate in the conversations between parent and staff, unless the parents or staff posed direct questions. The observations were carefully described in field notes, and where possible transcribed during the actual observation or directly after it. The next step consisted of reading the field notes, playing back the scenes in the mind and summarising the content as it appeared in its complexity, including personal reflections.

Sometimes observations were supplemented with interviews in order to deepen understanding. A total of thirty-nine interviews were performed: ten with parents (P), six with paediatric nurse assistants (PNA), eight with nurses (N) and fifteen with staff in management positions in the units (M). The management staff consisted of two operational managers, three unit managers, two assistant unit managers, two medical officers and six section managers, with between 0.5 and 19 years management experience (Md = 6). The participants were asked to reflect as openly as possible on their personal experiences of parental participation. After a situation had been observed, the parents, paediatric nurse assistants and nurses were asked an open question: What was your experience of the situation in the care room? The initial open question in the interviews with the management staff was: Which strategies did you use to facilitate parents' participation in the care of their child in this unit? Attendant questions posed were: Could you explain what you mean? Could you describe it in more detail? The aim of these open questions was to encourage the participants to talk more about and reflect on their experiences [20]. Some interviews with parents, paediatric nurse assistants and nurses were tape-recorded and transcribed word by word and some were carefully described in field notes. All the interviews with the management were tape-recorded and transcribed verbatim to text.

### Analysis

The analysis was based on texts from the observations and interviews, which were treated as one text based on principles described by Dahlberg et al. [19]. It was important that this lifeworld hermeneutic approach did not to use any predetermined hypotheses or any theories or other interpretive sources decided upon beforehand. Like all forms of text analyses, the interpretative analysis was a dialogue with the message of the texts [19] and was aimed at finding and comparing meanings. All the text was read openly and critically several times to find the meaning of the phenomenon, the hidden as well as explanations that were not immediately obvious. The analytic phase was thus open and flexible with a distancing, reflective and critical approach. The interpretations of the parts were constantly compared with the interpretation of the whole in order to decide whether there was a discrepancy between the understanding of the parts and the understanding of the whole [19,20]. Four interpretative themes of the conditions for parents' participation in the care of their child were identified and finally compared and put together in a new way in a "main interpretation" in order to understand further meanings of the phenomenon.

## Results

The four interpretative themes are presented below followed by the main interpretation of the phenomenon "conditions for parents' participation in the care of their child in neonatal intensive care".

### The care environment is dominated by medical technique

Two aspects of the care environment emerged as central, both of which facilitated and hindered parents' participation in the care of their children. These were the layout of the care rooms with their medical-technical equipment, and the specialisation of the care.

Both of the NICUs had two intensive care rooms, one had one light-care room and the other had two. An individual child could be transferred between these two types of rooms depending on the child's state of health. Other important rooms were the parents' rooms where they could stay, sometimes together with their child if the child's condition allowed it. One of the NICUs had enough such parent rooms but the other had only two.*"That there are no parents' rooms, you cannot then have such high expectations that they should participate in the care either." (N) *This led to a lot of practical problems if the mother was discharged from the maternity ward while the child was still being treated in the NICU. It forced parents to sleep at home and to come to the NICU daily, and it usually led to shorter stays as there were no rooms for the parents to rest and be in on their own. One father who had previously been allowed to stay in a parents' room expressed how it had improved his chances of being present: *"It was much better, you were with him more and it was easier to just go in." (P) *Neither of the units had joint care rooms where the recently delivered mother and ill child could be cared for together, and both staff and parents expressed a need for this form of care. For a mother who has just given birth, and is sometimes seriously ill, not to have the opportunity to rest in a bed but to be directed to sit on a chair made it more difficult to be present: "*I sat next to my child out of duty because I felt that I was really too tired, I just wanted to be in my bed, I couldn't cope." (P)*

The wards were a central part. At one of the units, they were quite spacious, though the technical equipment at each care place took a lot of space. At times it was cramped around the child with parents and staff sharing the space. "*There can be 20–25 persons on a ward and there is a lot of equipment and things." (N) *The staff made an effort to make a private sphere for the child's family around each care place with screens or curtains that were drawn, but the large number of people going through the ward, like a road junction, prevented the parents from being undisturbed with their children, because even with the curtains drawn or the screens around the care place, the noise could not be shut out. It seemed more stressful than calming for parents to be placed with their child in an environment with constant activity and loud equipment.

The activity on the wards varied with a peak in the morning, but at one of the units the working pace was constantly high with much overcrowding. At times, there was a shortage of staff at both units, which made it difficult for the parents to approach the staff. "*The problem every time you came was having to find staff; who was looking after my child." (P)*

The medical-technical care gave the care environment a special character that signalled the priorities and explained why the parents could feel "in the way" among the equipment and staff that surrounded their child. Nonetheless, the parents seemed to become used to it after a while and they started to act like the staff, such as turning off alarms themselves. Views on whether this type of parent participation was good or bad were divided among the staff. "*The parents do a lot, even with the equipment, and that terrifies me. That they pull the cables apart... the first times they are there to care for their child, someone (staff) might say, 'Yes you can take out these electrodes.' But that is when the staff is there; the next time the dad might turn off the alarm to the respirator." (N)*

The staff showed a high level of competence in emergency and intensive care of the child and appeared to prioritise this type of care over nursing care. *"If you are interested in equipment, tubes and leads, that is high status. If you are interested in meeting people, conversation, maybe it hasn't got as high a status." (M) *Presence and participation by parents at the NICU was often pointed out as central by staff, but the nature of the care environment made it less important. The medical-technical care had a clear place. This may seem obvious as it supports the survival and recovery of the children, but its profile meant that less obvious nursing care was pushed out, instead of the two being complementary parts of care characterised by a holistic view. As nursing care includes a welcoming approach to the parents of sick children, the interpretation follows that presence and participation by parents was not considered to have as high a priority as the medical-technical aspects of the care.

### The rounds focus on the medical diagnosis, while the caring needs are disregarded

The round played a central part in the care environment, but here it is presented as a separate theme as it played a very prominent role in the interpretation of conditions for parents' participation in the care of their child. At both units, the round routines reinforced the medical-technical emphasis on care. Every morning, a round was carried out, the time of which was determined by the physicians' other undertakings at the hospital. During the round, the discussion focused on the medical status of the child while its nursing care needs were considered to varying degrees. This might express itself in, for example, the nurse not always having knowledge of or paying attention to the family's social situation, with factors that affected the chances of being near the child. *Physician 1 asks: "How are the parents?" The nurse answers: "The father is on sick leave, but the mother is here." Physician 2 interposes: "The father is on long-term sick leave for depression and finds it difficult to care for the children. He can drop off and collect at the daycare centre and be at home alone with the three children for a maximum of 1 1/2 hours, so then the mother can be here." The nurse says: "Well, I knew the father was on sick leave, but I didn't know why." (O)*

At one of the units, parents were not allowed to be present during the round, even though the management thought it could be positive. The exclusion of parents from the round prevented their participation and created unnecessary worry for them, as expressed by one of the mothers as follows: *"At the time of the round, there was a total ban on entering the ward, and then you wonder as a parent why you can't listen when it is a round for your child?" (P) *This was in direct contrast to the other unit where the parents where invited and encouraged to take part in the round of their child:*"There is much greater consideration for the parents and focus on them participating, so it has improved a lot." (M) *Here the parents were seen as a resource as they could contribute valuable information about their child. It also saved the physicians' time as they could inform the parents directly of the medical state and care of their child. This seemed to ease the parents' worries, but they often had a low profile. Sometimes the physicians would ask if they wondered about anything, but they usually said no. Often the nurse would speak for the parents, for those who were absent as well as those who "were too tired" to ask their questions. The physician's time for each child and to inform the parents during the round was often very limited. Sometimes parents of other children were on the ward and could hear what was being said, even confidential information.*The physician opens the door to the corridor, turns round and asks the mother: "Is there anything else you wonder about?" The mother answers: "No, there isn't." The nurse who is standing by the mother says: "But you wondered before whether your child could have a funnel instead of an oxygen mask." The physician closes the door, goes back to mother and informs her of these two alternatives. (O) *After the round, it was usual for the nurse to go round to those parents who had been present and clarify what the physician had said.

At the unit where the parents were not allowed to be present during the round, the parents were not routinely informed afterwards either. This is how one new mother expressed herself:*"The nurses you asked referred to the physicians all the time, 'we'll see on the round in the morning,' but as we were never given any information after the round, it was still up in the air all the time." (P) *Naturally, this led to many questions remaining unanswered. Individual parent-physician talks on the state of the child were difficult to arrange at other times, and were often at the parents' own initiative. One mother who waited for more than a week to talk to a physician said:*"I was convinced that someone in neonatal would sit down and talk to me about how it had gone but no one did... then I wouldn't have had to be down on the maternity ward wondering what was happening and how he was, and I would have been calmer then." (P) *For a parent to have to demand to talk to a physician about the state and treatment of his/her child may seem strange as it is seen as a natural routine and right. At the unit where the parents were welcome to be present during the round of their child, there was greater access to talk to physicians, even though there was no big difference in the density of physicians at the two units to explain the differences between these contrasting routines.*"The physicians are here all day, so if you have any questions just make an appointment to talk to them." (N)*

### Participation is on the terms of the staff and the activity

The third theme looks at the way the attitude of the staff and the other activities affect the parents' conditions to participate in the care of their child at the NICU. It was considered professional to look after the individual needs of the parents, and by setting an example to colleagues; participation could be made easier for parents.*"I have a responsibility to make sure I tolerate the presence of the parents, showing the others that this is how we should work." (N) *In the many meetings observed between staff and parents, the manner of the staff stood out. There were many examples of how staff listened in and gave support, but unfortunately also shortcomings in flexibility to the parent.*The child lies in an incubator and lets out a whimper. The father turns the child, gives him the dummy and holds him with his hands. The child starts to scream and the paediatric nurse assistant comes up to them, looks at the child and says... "Little one should we turn you?" The father replies: "I have just turned him." The paediatric nurse assistant turns the child without saying anything. (O)*

When the parents arrived at the unit for the first time, they were usually received by the staff on the ward at which their child was cared for. They were informed of the medical condition of their child and the equipment to which their child was connected, given oral and written information about the routines at the unit, encouraged to be with their child whenever they wanted, and invited to take part in the care of their child: "*You can come whenever you like and help with all sorts of things, preferably everything; it is your child and although he is in an incubator that is no obstacle, we will help you." (PNA) *None of the units had a routine formal introduction talk despite the parents' needs and management's emphasis on the importance of such: *"I would like to sit down when I come up, that is, a real introduction talk with the physician and paediatric nurse." (P)//"I have tried for years for us to have a real introduction talk; it might be the first time you experience parenthood." (M)*

Mothers who had just given birth were usually taken to the unit by the staff from the delivery or maternity ward in a bed or wheelchair due to their medical condition or the distance between the units. Being restricted to going to and leaving the NICU based on staff availability to accompany them stood out as an obstacle to their presence and participation. When parents returned to the NICU, the staff usually took the time to tell them what had happened to their child since they were last there, but there were times when the parents were not given this attention.

"It felt as if I disturbed them when I entered." (P)//The door opens, a father looks in on the ward and says hello. The paediatric nurse assistant does not return his greeting. He takes the mother into the ward in her wheelchair, she gets out and the wheelchair is parked in a corner. The parents go to the washbasin and wash their hands, sit down with their child and try to make eye contact with the paediatric nurse assistant who does not look in their direction. (O)

The impressions of the invitations and expectations of parents to participate were ambiguous. The parents were often directly involved in feeding their child without even being asked, as shown by the following observation:*The parents are with their child for the first time and the child is going to be tube fed. The nurse connects the syringe to the tube and hands it to the father and says: "Maybe Dad would like to hold it?" The father backs off, takes the syringe in his hand and says, "Me? Ok, do I just hold it?" "Yes," says the nurse and goes off to check the infusion pumps. (O) *At other times, there was a lack of clarity and uncertainty among the staff of the extent to which requirements should be expressed to parents, and they did not routinely find out why, for example, a mother who was cared for on the maternity ward was absent for a whole "shift". There was also a lack of routines for, for example, documenting the presence of parents, which meant that many shifts could pass with no attempt being made to find out the reason for the absence. According to one nurse, the staff ought to be clearer on what was expected such as stressing the importance to a parent who was often absent of being present more of the time and taking part in the child's care. One explanation for this lack of clarity was a fear of making the parent feel guilty, and the welfare officer was often asked to "solve" this sensitive situation instead:*"We have someone who doesn't have a child already who only comes for one meal a day, but then maybe you have to find out why, if it's fear or... Then you can bring in the welfare officer to solve it, she can find out a little more on another level." (N) *This conflict of wanting to demand greater parent participation, a lack of routines for documenting the presence of parents and, at the same time, uncertainty of which demands could be made and how much involvement there should be in the family's social situation became an obstacle to parents' participation in the care of their child.

Parents of children who had been cared for at a NICU for a long time were asked more often how they wanted things to be: *"We would like to remove the navel catheter, but if you'd like we can wait with that so you can take her out of the incubator." (O) *The parents' wishes could also be ignored by first being invited to participate and then later not being given the opportunity to do so if it did not fit in with the activity. *The parents are with their five-day-old child, the mother for the second time, and the paediatric nurse assistant asks them: "Should I show you how to cup feed him and then you can do it yourselves?" She lifts the sleeping child and is about to start feeding him when the round enters the ward. The parents are shown into the corridor and the paediatric nurse assistant cup feeds the child. The child is put to bed, the round finishes and the parents come in. They go to their child and ask: "Is he going to eat?" The paediatric nurse assistant replies: "He has had some, exemplary baby. He ate by himself, really good, it went straight down." The parents go up to the child's bed and lift the cover. The paediatric nurse assistant says: "He is sleeping now, so we'll let him sleep." The parents sit down beside the bed but cannot see their child as the canopy is drawn and leave the unit after a couple of minutes. (O)*

Another activity that was adapted to the situation was the physician's discharge examination of the child and discharge talk to the parents, which were normally carried out on the ward. It usually entailed a quick medical examination of the child and a few minutes' talk with the parents. This was often followed by a longer talk with the nurse. The parents seemed to attach greater importance to this discharge talk than the opportunity they were given. One parent who questioned the format expressed the following: *"Then a physician came who I had never seen before and carried out a very quick paediatrician's examination, and then he said, 'yes, great, bye,' and then I lost the plot. 'What do you call this, where is the talk,' I said, which made him quite agitated with me, because I thought, 'is this what you call a discharge talk, then I don't know how you work here." (P) *The fast tempo of the discharge talk can be interpreted as the tasks waiting for the physicians having higher priority than the parents' needs for information and stands out as an obstacle to parent participation.

The professional role of the staff was undergoing change and a consultative role towards the parents was being worked out.*"Now the staff are being outmanoeuvred by the parents, going from being an expert to being a consultative adviser and supporter of the parents." (M) *There were also question marks about this new role,*"The parents have a natural, prominent role, but what is our role?" (N) *Parent participation was of benefit to the units. Parents were considered to relieve the staff in their work, support breast-feeding and reduce the duration of the child's care at the NICU as the parents got to know and care for their child better, *"... also of economic importance as our parents look after as much as they can." (M) *Even if it was considered to be beneficial that the parent took part in the care of their child, the constant presence of parents could be seen as tiring,*"... it's the ones who have really been parents to their children who are the tiring ones, because they have sat on a desk chair by their incubator and observed and questioned when people have done things in different ways with their child, and we have found that really hard work." (M) *It may seem like a challenge to find a professional role that is governed by cooperation with the participating parents, and not to see the parents in this "partnership" as competitors in the care of the child.

### Participation is important, but the economy is the controlling factor

A fourth theme describes the management's views on the parents' participation in the care of their child at the NICU. There was a high level of awareness of the importance of this and it was central to the goals of the activity, but there were no tangible guidelines as to how this should be done: *"We must create the conditions to allow them to participate and that means we must be able to offer the parents the chance to be here." (M) *The management saw it as its responsibility to promote an approach to care based on respect for the needs of the parents as well as the work of the staff. One necessary condition of developing neonatal intensive care was considered to be that the different categories of staff worked for the same goal, but in practice this was not the case. One reason was considered to be the difficulty of bringing together all the professional groups: the physician group was often not part of the unit's project.*"We miss the physicians in many situations when we discuss department routines or care routines." (M) *Another pattern noted by the management was that the staff started from their own needs when discussing care routines and this was believed to prevent the development of care at the NICU. In discussions on basic values at the unit, the staff were given the opportunity to reflect on their approach to parents in a more self-critical way.

"*Sometimes I have a feeling that we forget we are here for the patient, for the parents, maybe we are more used to thinking about ourselves, putting ourselves first." (M)// *"*We also have a shared responsibility for driving development at the neonatal unit, and sometimes maybe personal preferences have to take a back seat to the common good." (M)//"It's more about our attitude, we should get into our heads how we should behave and what our policy should be." (M)*

One way of getting parents to participate was to offer parental training, of which the management had seen positive effects. The unit nurses then talked to the parents as a group about different subjects such as what it means to be a parent of a child at a NICU. At one of the units, all the parents were invited to participate, while at the other unit only some parents were chosen. The fact that parental training was only offered to selected parents at one of the units may appear to be an expression of the management still not seeing its full value. The management at both units found that the parents who had participated in the activity took greater responsibility or became more active in their child's care.

The management was therefore greatly aware of the importance of drawing up strategies to promote parents' participation in the care of their child at the NICU, but this was subordinate to the primary goal of the activity, which was to adapt the care resources based on the order for care and the economic conditions set by the politicians. This higher goal became an obstacle to developing a care environment in which participation by parents could be improved. *"Having a balanced economy is the most important thing, as long as patient safety can be guaranteed. We are the implementers, but the politicians who decide what should be done with the tax payers' money; they represent the people." (M)*

### Main interpretation

A main interpretation emerges from the four themes interpreted above on the way conditions are created for parents to participate in the care of their child in neonatal intensive care. This expresses many contradictions regarding visions and goals and the prevailing reality of care. The management and staff both had high ambitions to develop ideal care that promoted parent participation in the care of their child at the NICU. In theory, they knew how they ought to behave, but observations showed that parents had limited opportunities to take the initiative and be active in the care, as individual staff decided, in a professional capacity, whether or not it was appropriate. Nursing care and the development of reliable and supporting relationships with the children's parents took on a secondary role. The lack of space for parents, shortage of staff, and recurrent overcrowding at one of the units made it more difficult for the staff to invite parents to care for their children. The format of the round at one of the units, where the parents were prohibited from being present, and the difficulty of communicating with physicians, conflicted with the invitation by the staff to the parents on their arrival at the NICU to participate in their child's care. Another contradictory aspect was the staff's expectations, on the one hand that parents should be present and participate in their child's care, and on the other their hesitance and insecurity of finding out the reasons for parents' absence. Staff in a management capacity, from section responsibility to operational responsibility at an overall level, expressed that the goal of the activity was to promote presence and participation by parents, and they dealt with this at development and training days etc. This too became a contradiction, as the economic resources were still the deciding force in the development of care at the unit.

## Discussion

All in all, the field study expresses that, in practice, there was no consistent basis of care values to guide participation by parents in the care of their child. The children "belonged" more to the ward than to the parents. This concurs with other studies [9,12,21,22]. At the two NICUs, the dominating medical-technical care was put against the nursing care. Medical-technical care was valued more highly; it was usually being carried out first, even in so-called non-emergency situations.

It is nothing new that emergency and intensive care environments have a medical-technical focus and that the advanced medical-technical equipment is visible, separating it from the "ordinary" ward. Another Swedish study has shown that staff at an emergency unit focused on carrying out medically advanced tasks at the expense of nursing care work [23]. One reason may be that it is completely unthinkable, and can also be punishable, to put aside medical-technical care, but the consequences of putting aside nursing care, which includes developing good relations between parents and carers, are more unclear. However, research has shown that the parents' lack of reliable relations in the care of children at the NICU can contribute to uncertain parent identity over a long period [7].

Parents are responsible for their children's needs for good care being met. According to the Swedish so-called Children and Parents Code, the parent represents the child and has a right and duty to decide on issues concerning the child when it is cared for in hospital [24]. In order to take this responsibility, however, the parent must be offered sensible conditions, e.g., being able to be with and receive information about the child at the NICU. It may seem strange that the parents at one of the units in the field study accepted being sent out into the corridor during the round, though it can be explained by it being difficult to demand access to a child in a critical situation, as the child requires highly specialised care. The parents were then in a very strong position of dependence on the staff who cared for their sick children, and they needed some kind of invitation to participate from the staff. The responsibility that should be taken by the parent ought to be made clear in a dialogue between the staff and parent.

Our result is similar to other research into parental participation in the paediatric care context, which has concluded that in order to identify and satisfy parents' needs, staff should initiate communication with parents [25-27]. The care of children at the NICU includes support for the child's parent. Staff often has clear ideas about what parents could be involved in and often take parent participation for granted [26,28]. The person who knows most about which kind of support is needed is the parent himself/herself, or to use words of philosopher Lögstrup [29]: "in order to help another person, we must let the person himself/herself decide what is most helpful", i.e., give the parent the opportunity to decide what he/she wishes to participate in.

The managers of the NICUs were unanimous in wanting the staff to operate the care with the participation of parents, and they arranged training days and discussions to raise awareness of the importance of this. For the staff to be aware of how the best care ought to be conveyed, however, and to be partly prevented from giving it due to the layout of the rooms, overcrowding and staff shortage, would probably lead to frustration. The management carries much of the responsibility for this frustration and should work to make the goals of participative parents possible. Being a skilful professional "carer" means having not only medical-technical skills but also nursing skills including creating good relations with parents. However, it emerged from the field studies that the meetings and invitations to the parents to take part in their child's care often failed. In an environment with care focused on the sick newborn child and with parents being faced with the fact that their child needs care at the NICU, the meeting and relationship with the staff is a necessary link between parent and child. The management has a responsibility to create tools that ensure quality of care and include allowing parents to participate in their child's care at the NICU. One way of doing this is to define staff guidelines on working with children's families [28].

### Methodological reflections

The research process used in this field study showed limitations and strengths. If the researcher is an expert in the area being studied, there is a risk he/she will forget his/her role as an observer and act like a nurse. The opposite can also happen. The study can be influenced by suspiciousness when a researcher coming from the "outside" is not able to understand what it is really all about [19]. The strength of this study, however, was that the first author was familiar specifically with caring and the complex environment in the NICU. Reflections on how this influenced the interpretation were necessary throughout the research process. An awareness of pre-understanding and openness, closeness and distance to the studied phenomenon was of importance and included the uniqueness of people's lifeworld and the complex environment in which the phenomenon took place. An objective understanding was not possible, but the cooperation between the three authors was an asset to this process [20], i.e. to understand more about the meaning of conditions for parents' participation in the care of their child.

## Conclusion

The result of the field study points to a number of contradictions in the way parents are given conditions to take part in neonatal intensive care. The goal of the activity was to promote presence and participation by the parent, but this was subordinate to the economic resources and the individual assessment by the staff of what is practically appropriate. Dominating medical-technical care, a shortage of staff and space for parents also made it more difficult for the staff to involve the parents and there were no tangible strategies to develop optimal conditions for parents to be present and involved. Furthermore, greater knowledge and understanding of the parents' conditions for participating in the NICU can create conditions for tangible measures such as the availability of parents' rooms and joint care of mother and child with the mother's bed next to the child.

## Competing interests

The author(s) declare that they have no competing interests.

## Authors' contributions

All the authors contributed to all the stages of the research, from planning to the final manuscript, except the data collection, which was performed by HW.

## Pre-publication history

The pre-publication history for this paper can be accessed here:


